# The oral microbiome of children in health and disease—a literature review

**DOI:** 10.3389/froh.2024.1477004

**Published:** 2024-10-22

**Authors:** Salma G. AlHarbi, Abdullah S. Almushayt, Shatha Bamashmous, Turki S. Abujamel, Nada Othman Bamashmous

**Affiliations:** ^1^ Pediatric Dentistry Department, Faculty of Dentistry, King Abdulaziz University, Jeddah, Saudi Arabia; ^2^ Dental Department, Ministry of Health, Yanbu, Saudi Arabia; ^3^ Department of Periodontology, Faculty of Dentistry, King Abdulaziz University, Jeddah, Makkah, Saudi Arabia; ^4^ Division of Vaccines and Immunotherapy, King Fahd Center for Medical Research, King Abdulaziz University, Jeddah, Makkah, Saudi Arabia; ^5^ Special Infectious Agents Unit, King Fahd Center for Medical Research, King Abdulaziz University, Jeddah, Makkah, Saudi Arabia

**Keywords:** children, caries, dental plaque, gingivitis, periodontitis, oral microbiome, oral cancer

## Abstract

The oral microbiome is a complex community of microorganisms residing in the oral cavity interacting with each other and with the host in a state of equilibrium. Disruptions in this balance can result in both oral and systemic conditions. Historically, studying the oral microbiome faced limitations due to culture-dependent techniques that could not capture the complexity and diversity of the microbial community. The emergence of advanced genomic technologies and the ease of sample collection from the oral cavity has revolutionized the understanding of the oral microbiome, providing valuable insights into the bacterial community in both health and disease. This review explores the oral microbiome in children, discussing its formation and dynamics in both states of health and disease, its role in various conditions such as dental caries, periodontal disease, oral cancer, cleft lip and palate, and explores its connection to several systemic consequences.

## Introduction and background

First introduced by Joshua Lederberg, the human microbiome represents an ecosystem of microorganisms that inhabit the human body and interact with each other in a symbiotic way (1). The current microbiome reflects millions of years of coevolution between humans and microorganisms. Over time, both have mutually adapted to each other, and the microbiome now plays an essential role in our health and disease (2). To research host-microbe interactions, traditional microbial ecology methods have relied on culture-dependent methodologies which led to a limited perspective of microbiota (3). These methods rely on cultivating microorganisms in laboratory settings, enabling in-depth analysis of their biochemical and physiological traits, such as metabolic functions and resistance to antibiotics. While these methods have been fundamental in microbiology, they face notable limitations, particularly in detecting anaerobic microbes that cannot easily grow in standard lab environments. Research indicates that traditional culturing techniques can overlook a large portion of the oral microbiome, with estimates suggesting that more than 50% of oral bacteria are “unculturable” using conventional approaches (4). Molecular technologies, such as next-generation sequencing, enabled comprehensive investigation of the microbes in the different body habitat, including those that cannot be cultured in a laboratory. This approach has led to a revolution in understanding microbial communities, allowing researchers to explore the composition, diversity, and interactions of these communities in their natural habitat (3). However, these techniques also have limitations, including higher costs, the need for advanced bioinformatics expertise, and the inability to directly measure microbial viability or activity as culture-based studies do (5).

The oral cavity represents the second most diverse microbial community in the human body after the gut, harbouring over 700 different types of bacteria (6). These bacteria inhabit various locations in the mouth, including the teeth, tongue, periodontal structures, and cheeks; all are interconnected by saliva (7). The human oral microbiome was reported to be influenced by different factors from maternal and environmental sources. The interactions occurring between the microbiome and the human host during early life are accountable for shaping innate and acquired immune functions, as well as physiological development, which in turn have implications for future health outcomes (8).

Most of the literature on the oral microbiome focuses on adults, detailing both its composition in healthy individuals and its alterations in various pathological conditions, in contrast, the studies exploring the microbiome of children is relatively sparse, with a predominant focus on dental caries (9). This review aims to highlight the current state of knowledge regarding the microbiome of children in both health and disease, state gaps in current understanding, and propose future research directions.

## Materials and methods

A literature search was conducted across several electronic databases, including PubMed/MEDLINE, Scopus, the COCHRANE library and Web of Science. The search was performed using a combination of relevant keywords and MeSH terms such as “oral microbiome”, “oral microbiota”, “oral bacteria”, “children”, “pediatric oral health”, “dental caries”, “periodontal disease”, “Molar-Incisor Hypomineralization”, “oral cancer”, “cleft lip and palate”, “systemic disease”, “diseases”. Only articles published between January 1990 and January 2024 in English have been included.

The search obtained 345,163 results, after the screening of the titles and abstracts, non-topic entries were excluded. References were exported and managed using EndNote 20.

Due to the extensive number of articles included and the diverse methods and outcomes among the identified studies, it was not feasible to present the findings as a systematic review or meta-analysis. As a result, a narrative review was carried out.

## Acquisition and establishment of the oral microbiome

A pregnant woman's oral health and oral microbiome can have a direct impact on her pregnancy and her developing baby. Additionally, it is proposed that the immune system of the fetus develops a prenatal tolerance to the maternal microbiome considering it safe during postnatal exposure to and potentially contributing to the successful development of a balanced microbiome (10). Once a healthy microbiome is established, it must constantly adapt to changes in the oral environment, including changes in temperature, pH, forces of brushing and mastication, and the presence of different types of food and oral hygiene products (10).

At birth, infants encounter their mother's microbiome via vertical transmission. Vaginally born babies are exposed to their mother's vaginal and rectal microbes, while caesarean section babies are exposed to their mother's skin and hospital environment microbes (11, 12). This difference in exposure can have a significant impact on the development of the baby's microbiome. At the age of 3 months, vaginally born infants exhibit higher diversity of the microbiome at the taxonomic level (13). A study comparing maternal factors in the acquisition of *Streptococcus mutans* in infants revealed that infants born with caesarean section acquired *S. mutans* almost 12 months earlier compared to vaginally born infants (14). Diet is also reported as a factor affecting the microbiome, breast-fed infants possess oral *Lactobacillus* spp. that are absent in formula-fed infants at 3 months of age both *in vivo* and *in vitro* (15, 16). It has been proposed that microorganisms are obtained during a specific period during which colonization of certain bacteria occurs, known as “window of infectivity”. In the case of *S. mutans*, its colonization occurs between 19 and 31 months of age (17). An additional suggested “window”, occurring between the ages of 6 and 12 years, aligns with the emergence of permanent dentition (18).

In addition to vertical transmission, the oral microbiome can also be shared among individuals living in the same environment, such as siblings, this is termed horizontal transmission which contributes to the diversity of the oral microbiome (19, 20). This transmission may occur due to shared use of toys, foods, or utensils, potentially influenced by hygiene practices and awareness in these environments (21).

## Microbial diversity in the healthy oral cavity

Analysis of the composition of the healthy oral microbiome in infants, children, and adolescents based on 16S rRNA gene sequencing techniques using plaque and saliva samples found that *Proteobacteria*, *Fusobacterium*, *Actynobacteria*, *Bacteroidetes*, *Firmicutes*, *Synergistetes*, *Tenericutes*, *Capnocytophaga*, *Neisseria*, *Sreptococcus*, *Kingella*, *Leptotrichia*. *Burkholderia*, and *Strenotrophomas Enterobacteriaceae* are dominant genera with a high level of abundance at 12–24 months old. On the other hand, *Firmicutes*, *Proteobacteria*, *Actinobacillus*, *Bacteroidetes*, *Fusobacterium*, *Streptococcus*, *Prevotella*, *Veillonella*, *Neisseria*, *Rothya*, and *Haemophilus* are predominant genera in children (9).

In a cohort of 1-year old infants with a healthy oral cavity, a notable difference in the oral microbial composition was identified between children who maintained a caries-free status and those who developed cavitated caries. The relative abundance of *Prevotella nanceiensis*, *Leptotrichia sp. HMT 215*, *Prevotella melaninogenica*, and *Campylobacter concisus* was found to be significantly higher in the group of children who remained caries-free. This observation suggests a potential association between the prevalence of these specific microbial species and a reduced likelihood of developing cavitated caries in the studied population (22).

A multicenter longitudinal study exploring the establishment of a healthy oral microbiome in caries-free children aged 1–4 years, based on a sample of 119 individuals, the microbial composition in saliva and plaque experienced significant alterations from the age of 1–2.5 years, followed by more subtle microbial changes up to the age of 4 years. The study identified a general increase in microbial diversity as age increased, with limited number of taxa, including various species of the *Streptococcus* and *Gemella* genera, were consistently present in all samples across different time points in children. The study concluded that the oral ecosystem of caries-free children is characterized by significant heterogeneity and dynamic changes (23). By adolescence, puberty brings significant hormonal changes that influence the oral microbiome. Increased levels of sex hormones (e.g., estrogen, progesterone) create an environment conducive to the growth of *Porphyromonas* spp. and *Prevotella* spp. associated with gingival inflammation and periodontitis (24) (Table 1).

**Table 1:** Comparative summary of microbiome dynamics across different Age groups.

Age group	Key microbial communities	Key influences	Health implications
Infants	*Streptococcus salivarius*, *Lactobacillus spp.*	Mode of delivery, breastfeeding	Early establishment of beneficial microbes
Toddlers	*S. mutans*, *Gamella* spp. and *Fusobacterium* spp.	Teething, diet, oral hygiene, horizontal transmission	Susceptibility to early childhood caries, increased microbial diversity
Adolescents	*P. gingivalis*, *Prevotella* spp., *A. actinomycetemcomitans*	Hormonal changes, diet	Periodontal inflammation, greater risk of gingivitis and caries

Studies have also compared the oral microbiome between primary and permanent dentition, particularly focusing on the transitional period of mixed dentition. During this stage, significant differences in microbial composition are observed between primary and permanent teeth within the same oral cavity. One study found that permanent molars tend to have a distinct microbial profile compared to primary teeth, with variations in the abundance of specific bacterial genera including *Fusobacteria* and *Bacteroidetes*. These differences are influenced by the physicochemical and developmental changes in the oral environment during the eruption of permanent teeth, such as oxygen levels (25). Another study revealed that the transition from primary to permanent dentition is accompanied by increased microbial diversity. As it leads to the colonization of additional microbial species from genera such as *Streptococcus*, *Gemella*, *Granulicatella*, and *Veillonella*. As well as expanded functional capacities within the oral microbiome. These changes reflect the oral microbiome's adaptation to new niches created by the eruption of permanent teeth (26).

## Microbiome of children with dental caries

Dental caries is a complex disease caused by the interaction of four main factors, the susceptible host, the microbiome, the substrate, and the element of time **(**27). It is important to highlight that *S. mutans*, the extensively researched species linked to caries, comprises merely <1% of the overall bacterial community (28). As a result, identifying the microorganisms causing dental caries has been a significant focus of research for decades, and developments in the field of oral microbiome identification and analysis techniques have resulted in changes in the documented composition and characteristics of oral microbiota (29). Caries can develop in the absence of *Streptococcus* and *Lactobacillus* species*,* in addition, the *S. mutans* count has been found to be low even when caries is present, suggesting that other species may be responsible for caries development and progression (30). Besides *S. mutans*, various other species associated with caries were reported in the literature, including but not limited to species such as *Veillonella dispar, Prevotella* spp., *Lactobacillus* spp., *Leptotrichia* spp., *Actinomyces* spp., *Neisseria* spp., *Porphyromonas* spp., *Treponema* spp., and *Streptococcus sobrinus* (31–41).

When comparing microbial composition, the unstimulated saliva of children affected by severe early childhood caries with that of caries-free children, saliva of caries-free individuals showed elevated quantities of *Capnocytophaga* and *Leptotrichia* while levels of *Porphyromonas* and *Neisseria* were lower at genus level (34).

## Microbiome of children with molar incisor hypomineralization (MIH)

As alterations in the microbiome can contribute to oral disease, certain conditions can, in turn, induce changes in the microbiome, thereby influencing the oral health. MIH is one of those unique conditions in which microbiome is altered and caries progress faster in the affected teeth compared to none-affected teeth (42). Molar-incisor hypomineralization (MIH) is defined as a developmental enamel defect that affects at least one permanent first molar. Affected anterior teeth might also be observed (43). Caries can easily develop in the affected teeth due to their porous nature, and this problem is exacerbated by the fact that children avoid cleaning their sensitive teeth, resulting in greater food and biofilm stagnation (43). The study by Hernández et al. observed more bacterial diversity, higher count of bacterial niches, and predominance of proteolytic bacterial genera such as *Catonella, Fusobacterium, Campylobacter, Tannerella, Centipeda, Streptobacillus*, and *Alloprevotella* in teeth affected with MIH, suggesting the presence of a unique microbiome related to MIH (42).

## Microbiome of children with periodontal disease

The term “periodontal diseases” encompasses both inherited and acquired disorders affecting the tissues that surround and support the teeth, including the gingiva, cementum, periodontal ligament (PDL), and alveolar bone (44). The evidence suggests that the development of periodontal diseases is associated with an increase in the quantities of Gram-negative bacteria and anaerobes in subgingival plaque (45, 46). Gingivitis is very common in children and adolescents (47, 48) and can progress to more severe forms of periodontal disease, such as periodontitis, involving the loss of connective tissue and bone around the teeth (12, 49–51). A systematic reviews on the relationship between maternal periodontitis and adverse pregnancy outcomes concluded that mothers with periodontal disease face a higher risk of preterm birth, delivering a low-birth-weight baby, and developing preeclampsia (52). It is suggested that periodontal pathogens or their by-products, such as endotoxins, reach the placenta and the fetus (53).

Several studies have investigated the microbiome of periodontal disease in children. The main evidence related to a particular microbial cause of periodontitis in children is derived from research on a distinct clinical periodontal syndrome that impacts adolescents exhibiting a pattern of lesions distributed around molars and incisors, known as localized juvenile periodontitis (LJP). In LJP, *A. actinomycetemcomitans*, *Capnocytophaga sp*., *Eikenella corrodens*, *Prevotella intermedia*, and motile anaerobic rods, such as *Campylobacter rectus* are the most detected organisms (54). *A. actinomycetemcomitans*, *Capnocytophaga sp*., and *P. intermedia* have been commonly identified as well in the subgingival microbiota of periodontitis in the primary dentition in children (55–60). A recent study found that schoolchildren with early-onset periodontitis had a higher abundance of certain bacteria, such as *Campylobacter* species, *Prevotella intermedia*, and *Fusobacterium nucleatum*, *Porphyromonas gingivalis*, *Treponema denticola*, and *Tannerella forsythia* (61).

## Microbiome of children with oral cancer

A developing concept in the field of cancer biology suggests that the microbiome serves as a significant environmental factor that influences the process of oncogenesis. There is a growing body of evidence indicating a connection between alterations in the human microbiome and specific types of cancer (62–65). Specific oral taxa, such as *Porphyromonas gingivalis* and *Fusobacterium nucleatum*, possess carcinogenic characteristics, including the inhibition of apoptosis, stimulation of cellular proliferation, initiation of chronic inflammation, facilitation of cellular invasion, and the generation of carcinogenic substances (66). Both of the aforementioned taxa have the capacity to release endotoxins, specifically lipopolysaccharides, which, in turn, can trigger the production of cytokines associated with inflammation, which is a primary factor in bacteria-induced inflammation and serves as a contributing factor to the process of carcinogenesis as well (67–69). A novel study investigated the oral microbiome of children diagnosed with solid tumors, analyzing diversity, composition, and gene profiles using saliva samples. Children with tumors exhibited a reduction in oral microbiome diversity compared to the healthy controls, with genera such as *Veillonellaceae, Firmicutes unclassified, Coriobacteriia, Atopobiaceae, Negativicutes* significantly enriched among them. This study suggests that oral microbiome could function as a non-invasive diagnostic tool for patients with pediatric solid tumors (70).

Each year, a significant number of newly diagnosed cancer cases are reported, with oral cancers exhibiting a particularly high prevalence among them, specifically oral squamous cell carcinoma (OSCC) (71). While OSCC is very rare in children, studies primarily focused on the adult population have shown variations in the microbiome between a healthy oral cavity and OSCC. Although the specific pathogenic bacteria or bacterial spectrum linked to OSCC has not been determined (72), elevated concentrations of *Peptostreptococcus* spp., *Fusobacterium* spp., *Prevotella melaninogenica*, *Porphyromonas* spp., *Veillonella parvula, Haemophilus* spp., *Rothia* spp., and *Streptococcus* spp. have been identified in samples of OSCC (73–77).

## Oral microbiota and systemic diseases

The theory suggesting a connection between oral microbiome and systemic diseases revolves around the potential consequences of bacteremia, which denotes the presence of bacteria in the bloodstream, dental procedures as well as routine practices such as eating, chewing, or brushing can trigger bacteremia (78). Usually the immune system promptly manages and eliminates microorganisms from the systemic circulation, however, in specific cases, particularly in individuals with compromised immune systems, oral organisms may persist without elimination, colonizing certain distant sites and increasing the likelihood of systemic disease development (79). It was also found that certain systemic bacteria can reside in specific oral locations, such as: *Hemophilus influenzae*, *Pseudomonas aeruginosa*, and *Trophyrema whipplei* have been identified in the gingival sulcus (79). Conversely, oral bacteria such as *Porphyromonas gingivalis*, *Treponema denticol*a, and *Campylobacter rectus* have also been detected in systemic sites, including atheroma plaques, valvular vegetations, joint cavities, and the pancreas (79). Moreover, it is not just bacteria that can enter the bloodstream; their bacterial byproducts and endotoxins can also be discharged into the systemic circulation. This has the potential to initiate inflammatory reactions in particular locations, thereby heightening the risk of developing systemic diseases (80). On the other hand, systemic diseases can significantly influence the composition and dynamics of the oral microbiota, this is attributed to the high level of inflammation associated with these conditions affects the oral microbiota, leading to significant alterations (81).

Many studies have been conducted to examine the oral microbiome of children with systemic diseases. Francavilla et al. studied the salivary microbiome and metabolome in 13 children with celiac disease (CD) following gluten-free diets (T-CD) and their healthy counterparts (HC). The findings indicate an association between celiac disease and dysbiosis in the oral microbiota, potentially influencing the oral metabolome. Those with T-CD exhibited a less diverse salivary microbiome, with increased abundance of *Rothia* spp., *Porphyromonas endodontalis, Gemella* spp., *Prevotella nanceiensis, S. sanguinis*, and *Lachnospiraceae* spp. compared to their healthy counterparts. Furthermore, children with T-CD showed a reduced abundance of *Actinomyces species, Atopobium species,* and *Corynebacterium durum* (82). This is attributed to the fact that patients with CD typically exhibit lower levels of amylase and secretory IgA and IgM in their saliva (83, 84). Additionally, their saliva tends to have reduced buffering capacity, lower salivary flow rates, and lower concentrations of calcium, as well as decreased calcium/ phosphate ratios (85, 86).

Recent studies have revealed a link between neuropsychiatric disorders (NPD), such as autism spectrum disorder (ASD) and the gut microbiota, which can interact with and impact the brain via the Gut-Brain Axis (GBA). Similarly, there is an emerging concept of oral-microbiota-brain axis (OMBA). Research on the oral microbiome and its relationship with the brain indicates that microbes in the mouth may also play a role in influencing NPD outcomes (87). Oral microbiota can enter the brain through the cardiovascular system, where they are believed to directly contribute to the disruption of essential neurological functions and the deterioration of brain tissue due to the buildup of virulent byproducts (88). *P. gingivalis* is known to enter the bloodstream and reach the brain, where it establishes colonies and secretes neurotoxic proteases known as gingipains. These gingipains play a role in disrupting the processing of the transmembrane protein Amyloid Precursor Protein (APP), which is crucial for maintaining synaptic stability, as well as for promoting neuronal growth and protection (88).

Hicks et al. studied the oral microbiome alterations in children with ASD, they detected alterations in the salivary microbiome among children in the age range of 2–6 years, categorized into three developmental profiles: autism spectrum disorder (ASD; *n* = 180), nonautistic developmental delay (DD; *n* = 60), and typically developing (TD; *n* = 106) children (89). In the comparison of taxa between children with ASD and TD children, there was an increased abundance of two species in ASD children: *Limnohabitans sp. 63ED37-2* and *Planctomycetales* (89). The findings of the study suggest that disturbances in the gut microbiome observed in ASD may also extend to the oropharynx. Subsequently, the regular evaluation of children's oral microbiome could potentially be developed as a non-invasive and sensitive tool for diagnosing ASD and monitoring its progression (89).

Several studies have confirmed a relationship between diabetes mellitus (DM) and periodontitis in adults, as DM induces changes in connective tissue metabolism, leading to a decreased ability to resolve inflammation and undergo remodeling, which, in turn, exacerbates periodontal damage (90). However, studies in children are still limited. A cohort study conducted in 2021 analyzed the salivary microbiome of 37 children aged 5–15 years diagnosed with type 1 DM, in children with type 1 DM, *Streptococcus* genus was found to be more prevalent. In addition, 22 taxa at the genus level and 33 taxa at the species level were absent in the control group, whereas the control group showed 6 taxa at the genus level and 9 taxa at the species level that were not present in the diabetes group (91)*.* A cross-sectional study conducted in the same year evaluated the composition and abundance of bacterial microbiota in the oral swabs of children aged 10–18 years diagnosed with type 1 DM (well controlled and poorly controlled), compared with those of healthy children. The group of children with well controlled DM exhibited a notably elevated count of bacteria belonging to the *Streptococcus* genus compared to healthy children. The presence of *Streptococcus mitis* was notably higher in the group of children diagnosed with type 1 DM compared to their healthy counterparts, which is considered one of the primary colonizers of the pellicle (92). A recent study assessed the relationship between the composition of oral bacteria in saliva samples, oral hygiene practices, and glycemic control in a group of children with a mean age of 12.6 years diagnosed with type 1 DM. A high abundance of bacteria related to dental caries and periodontal disease, specifically, *Actinomyces* spp., *Aggregatibacter actinomycetemcomitans*, *Prevotella intermedia*, and *Lactobacillus* spp. were identified in all subjects. *S. mutans* was detected in approximately half of the samples, particularly among patients exhibiting poor glycemic control. Positive oral hygiene behaviors, such as regular toothbrush replacement and professional dental cleanings, were inversely correlated with the concurrent presence of *Tannerella forsythia*, *Treponema denticola*, and *Porphyromonas gingivalis* bacteria associated with periodontal disease (93). In children with DM, the formation of advanced glycation end products (AGEs), which occurs through a non-enzymatic reaction between sugars and proteins, lipids, or nucleic acids, is accelerated as a result of chronic hyperglycemia (91). AGEs disrupt the normal function of nearly all body organs including the oral cavity by triggering apoptosis, inflammation, protein dysfunction, mitochondrial impairment, and oxidative stress (94). It was also found that the periodontal pathogen *Tannerella forsythia* produces methylglyoxal (a precursor of AGEs) in gingival tissues, this finding suggests bidirectional relationship between periodontal disease and poor glycemic control (95).

## Microbiome of children with cleft lip and palate

Cleft lip and/or palate (CLP) is one of the conditions that has a significant influence on the composition of the oral microbiome. It is the most prevalent congenital craniofacial defect impacting the structure and function of the oral cavity and causing alterations in facial features (96–99). Children born with CLP may experience significant functional challenges related to routine activities such as sucking, swallowing, chewing, speaking, breathing, and social integration. Consequently, they necessitate extensive and prolonged rehabilitation starting from infancy and extending throughout adulthood (100–102).

A systematic review by Świtała et al. examined the current scientific literature on the oral microbiome in children with CLP, the review encompassed twelve studies investigating the microbiological status of individuals with CLP in comparison to non-cleft individuals (103). The overall findings revealed a higher incidence of caries in children with CLP compared to those without, associated with higher levels of *S. mutans* and/or *Lactobacillus* spp. (102, 104–111). Most of the studies suggested that this is attributed to various factors affecting the oral hygiene of those children, including, and not limited to: anxiety related to tooth brushing in the cleft area, lack of motivation, existence of malocclusion and structural irregularities, anomalies in tooth count, shape, and position, and prolonged presence of orthodontic appliances (103). The results of the studies presented in this review are shown in a summary table (Table 2).

**Table 2:** Summary of findings from studies on the oral microbiome of children in health and disease.

	Author, year	Age group (in years)	Main microbiota
Healthy oral cavity	D'Agostino et al. (9)	1–2	*Proteobacteria*, *Fusobacterium*, *Actynobacteria*, *Bacteroidetes*, *Firmicutes*, *Synergistetes*, *Tenericutes*, *Capnocytophaga*, *Neisseria*, *Sreptococcus*, *Kingella*, *Leptotrichia*. *Burkholderia*, and *Strenotrophomas* and *Enterobacteriaceae.*
		>2–15	*Firmicutes, Proteobacteria, Actinobacillus, Bacteroidetes, Fusobacterium, Streptococcus, Prevotella, Veillonella, Neisseria, Rothya,* and *Haemophilus.*
	Raksakmanut et al. (22)	1	*Prevotella nanceiensis*, *Leptotrichia sp. HMT 215*, *Prevotella melaninogenica*, and *Campylobacter concisus*
	Kahharova et al. (23)	1–4	*Streptococcus* and *Gemella*
Dental caries	Agnello et al. (31)	<6	*Veillonella HOT 780, Porphyromonas HOT 284, Streptococcus gordonii, Streptococcus sanguinis,* and *S. mutans.*
	Richards et al. (32)	2–7	*S. mutans*, *Scardo*via *wiggsiae*, *Parascardo*via *denticolens*, and *Lactobacillus salivarius.*
	Dashper et al. (33)	2 months–4	*S. mutans, S. sobrinus, V. parvula, Leptotrichia shahii, Scardo*via *wiggsiae and Leptotrichia IK040.*
	Hurley et al. (34)	<5	*Firmicutes, Bacteroidetes, Neisseria, Prevotella, Proteobacteria, Porphyromonas, S. mutans, Bifidobacterium* and *Scardo*via spp.
	Zheng et al. (35)	3–6	*S. mutans, Lactobacillus fermentum, Neisseria sica*, and *Veilonella dispar.*
	Ortiz et al. (36)	2–12	*Neisseria flavescens, Rothia aeria*, *Haemophilus pittmaniae, Lactococcus lactis*, *Selenomonas*, *Actinobaculum*, *Veillonella parvula*, and *Alloprevotella.*
	de Jesus et al. (37)	<6	*Veillonella dispar, S. mutans,* and *Neisseria.*
	Gussy et al. (38)	1 months–6	*S. mutans, Streptococcus sobrinus, Veillonella parvula, Leptotrichia shahi, Scardo*via *wiggsiae and Leptotrichia IK040*
	Qudeimat et al. (39)	7–9	*Leptotrichia shahii, Prevotella melaninogenica, Veillonella dispar, Leptotrichia HOT 498, and S. mutans.*
	Dinis et al. (40)	4–14	*S. mutans, Veillonella dispar, Streptococcus* spp., and *Prevotella* spp.
	Xu et al. (41)	3–6	*Streptococcus* spp., *Neisseria, Leptotrichia, Lautropia* and *Haemophilus*.
MIH	Hernández et al. (42)	6–12	*Catonella, Fusobacterium, Campylobacter, Tannerella, Centipeda, Streptobacillus,* and *Alloprevotella.*
Periodontal disease	Tonetti and Mombelli (54)	5–11	*A. actinomycetemcomitans*, *Capnocytophaga sp*., *Eikenella corrodens*, *Prevotella intermedia*, and *Campylobacter rectus.*
	Asikainen et al. (55)	10	A. actinomycetemcomitans
	Dibart et al. (56)	7	*Prevotella intermedia, Selenomonas noxia, Fusobacterium nucleatum, and Actinobacillus actinomycetemcomitans*
	Kammaet al. (57)	11	*Porphyromonas gingivalis, Prevotella intermedia, Capnocytophaga sputigena, Capnocytophaga ochracea,* and *Actinobacillus actinomycetemcomitans.*
	Sixou et al. (60)	4	*Actinobacillus actinomycetemcomitans,* and *Porphyromonas gingivalis.*
	Piwat et al. (61)	12–18	*Campylobacter* species, *Prevotella intermedia*, and *Fusobacterium nucleatum*, *Porphyromonas gingivalis*, *Treponema denticola*, and *Tannerella forsythia*.
Oral cancer	Cui et al. (70)	1–7	*Veillonellaceae, unclassified Firmicutes, Coriobacteriia, Atopobiaceae,* and *Negativicutes.*
CD	Francavilla et al. (82)	8.6–11.4	*Rothia, Porphyromonas endodontalis, Gemellaceae, Prevotella nanceiensis, S. sanguinis*, and *Lachnospiraceae.*
ASD	Hicks et al. (89)	2–6	*Limnohabitans sp. 63ED37-2* and *Planctomycetales.*
DM	Moskovitz et al. (91)	5–15	*Streptococcus* spp.
	Pachoński et al. (92)	10–18	*S. mitis*
	Carelli et al. (93)	10.4–14.8	*Actinomyces* spp., *Aggregatibacter actinomycetemcomitans*, *Prevotella intermedia*, *Lactobacillus* spp. and *S. mutans.*
CLP	Świtała et al. (103)	3–21	*S. mutans* and *Lactobacilli*

MIH, Molar-incisor hypomineralization; CD, celiac disease; ASD, autism spectrum disorder; DM, diabetes mellitus; CLP, cleft lip and/or palate.

## Ethical considerations in pediatric oral microbiome research

When addressing ethical considerations in pediatric oral microbiome research, several factors must be considered: First, informed consent and assent, which is typically obtained from parents or legal guardians, as children are generally not legally capable of providing full consent. However, ethical standards recommend also seeking assent from the child, depending on their age and cognitive development. This approach involves explaining the research in a way that is understandable for the child, ensuring they comprehend the study, and respecting their decision to participate or decline (112, 113). Second, sample collection, which is usually non-invasive (e.g., saliva, swabs) and cause minimal discomfort. Researchers should also ensure the child's understanding and comfort with the procedures. Any invasive methods must be justified by clear benefits outweighing the risks and discomfort (113). Third, privacy and data protection, which is a key concern, particularly when managing genetic or sensitive health data. Adherence to data protection laws, such as the General Data Protection Regulation (GDPR) in Europe and the Health Insurance Portability and Accountability Act (HIPAA) in the U.S., is essential. Researchers must ensure that personal data is anonymized and securely stored to protect children's privacy during and after the research (113).

## Conclusion

After years of investigation, researchers have progressively unveiled a novel understanding of the microbiome's involvement in both health and disease. It is now established that there is a bidirectional relationship between the host and its microbiome, as the microbiome can influence nearly every aspect of the host, and disturbances in its balance are linked to a broad spectrum of diseases, certain conditions can prompt alterations in the microbiome, consequently impacting oral health. Advanced research technologies facilitate the close examination of how the microbiome contributes to human health and participates in the development of diseases. However, the predominant focus of microbiome studies has been on the bacterial component, leaving the roles of fungi, viruses, and other microbes uncertain. Moreover, although dysbiosis of the microbiome is frequently observed in disease states, establishing the causative role of the microbiota remains an ongoing challenge. Consequently, numerous questions persist in this field, awaiting further exploration and clarification.

The future application of the oral microbiome for enhancing human health will rely on additional validation of microbial biomarkers specific to diseases. These markers need to be integrated into diagnostic and preventive programs that are not only sensitive and specific but also provide rapid results and are cost-effective for widespread implementation. When combined with human genomics, proteomics, transcriptomics, metabolomics, and utilization of artificial intelligence, the oral microbiome of children has the potential to play a central role in the advancement of precision medicine, facilitating the development of personalized preventive dental programs in the future.
